# The effects of a physical activity counseling program after an exacerbation in patients with Chronic Obstructive Pulmonary Disease: a randomized controlled pilot study

**DOI:** 10.1186/s12890-015-0126-8

**Published:** 2015-11-04

**Authors:** Miek Hornikx, Heleen Demeyer, Carlos Augusto Camillo, Wim Janssens, Thierry Troosters

**Affiliations:** Department of Cardiovascular Diseases, University Hospitals Leuven, KU Leuven-University of Leuven, B-3000 Leuven, Belgium; Department of Respiratory Diseases, University Hospitals Leuven, KU Leuven-University of Leuven, B-3000 Leuven, Belgium

**Keywords:** COPD, Exacerbation, Physical activity counseling, Real-time feedback

## Abstract

**Background:**

In some patients with COPD, the disease is characterized by exacerbations. Severe exacerbations warrant a hospitalization, with prolonged detrimental effects on physical activity. Interventions after an exacerbation may improve physical activity, with longstanding health benefits. Physical activity counseling and real-time feedback were effective in stable COPD. No evidence is available on the use of this therapeutic modality in patients after a COPD exacerbation.

**Methods:**

Thirty patients were randomly assigned to usual care or physical activity counseling, by telephone contacts at a frequency of 3 times a week and real-time feedback. Lung function, peripheral muscle strength, functional exercise capacity, symptom experience and COPD-related health status were assessed during hospital stay and 1 month later.

**Results:**

Both groups significantly recovered in physical activity (PAsteps: control group: 1013 ± 1275 steps vs intervention group: 984 ± 1208 steps (*p* = 0.0005); PAwalk: control group: 13 ± 14 min vs intervention group: 13 ± 16 min (*p* = 0.0002)), functional exercise capacity (control group: 64 ± 59 m (*p* = 0.002) vs intervention group: 67 ± 84 m (*p* = 0.02)) and COPD-related health status (CAT: control group: −5 [−7 to 1] (*p* = 0.02) vs intervention group: −3 [−10 to 1] points (*p* = 0.03)). No differences between groups were observed.

**Conclusion:**

From our pilot study, we concluded that telephone based physical activity counseling with pedometer feedback after an exacerbation did not result in better improvements in physical activity and clinical outcomes compared to usual care. Because of the difficult recruitment and the negative intermediate analyses, this study was not continued.

**Trial registration:**

Clinicaltrials.gov NCT02223962. Registered 4 September 2013.

## Background

Chronic Obstructive Pulmonary Disease (COPD) is characterized by exacerbations. These episodes of worsening of symptoms beyond the expected daily variation lead to substantial morbidity and mortality. Exacerbations accelerate disease progression and they have a negative impact on the quality of life of patients [1–5]. Severe exacerbations of COPD require a hospitalization, imposing a real burden on the socioeconomic system [1, 4]. During the hospital stay, patients are severely inactive, with a repercussion on their muscle strength and exercise capacity [6]. Physical activity (PA) levels of these patients are below the levels found in stable patients with COPD, even 1 month after discharge [7]. Immediately after their return home, patients experience several barriers to engage in PA, such as anxiety for dyspnea, the need for oxygen and environmental factors such as the weather [8]. Nevertheless, sustaining a physically inactive lifestyle increases the risk for a new hospital admission [9], increases mortality rates [9, 10] and has a negative impact on the onset and progression of comorbid conditions [11, 12]. These findings warrant the measurement and enhancement of PA after hospital discharge. So far, studies concentrated on formal pulmonary rehabilitation to improve physical activity after exacerbations, with inconsistent effects [12]. From the 10 studies [13–22] that have been published on this topic, 4 studies [17–19, 22] showed an increased level of physical activity after pulmonary rehabilitation. Furthermore, such programs may suffer from poor uptake, adherence and lack of accessibility [23]. Only 34 % (range 18 to 67 %) of eligible patients eventually participate in pulmonary rehabilitation [23–27]. Therefore, there is an urgent need for other interventions to promote physical activity after an exacerbation. In stable patients with COPD, nordic walking [28] was investigated and was found to be successful. PA counseling combined with real-time feedback has proven its effect in improving PA in healthy subjects [29], in patients with heart failure [30, 31], diabetes [11] and in stable patients with COPD. The use of the latter treatment modality has not been explored in patients immediately after a hospitalization for an acute exacerbation of COPD [32–34]. For this pilot study, we hypothesized that, through frequent PA counseling and real-time feedback, patients would potentially increase their PA level more rapidly in the month after an exacerbation compared to subjects in a control group, receiving usual care.

## Methods

### Subjects

Patients with COPD, hospitalized for an exacerbation of COPD were informed about the study and were included when the following inclusion criteria were met: 1) Male/female > 40 years of age 2) Diagnosis of COPD, defined as FEV_1_/FVC < 70 % (post-bronchodilation) 3) Hospitalized for a COPD exacerbation 4) Ability to work with electronic devices. Patients were excluded if they participated in pulmonary rehabilitation prior to the index hospital admission (and would return to the program after discharge) or suffered from a neurological or musculoskeletal disease that would prevent them from being active. The protocol was approved by the local medical ethics committee from the University Hospitals KULeuven and all patients gave their written informed consent.

### Sample size calculation

The sample size calculation was performed using G*Power (version 3.1.6). Based on previous research, we assumed the intervention group to reach a walking time that equals the walking time in stable patients with COPD (44 ± 20 min) [35]. The control group was estimated to achieve 28 ± 20 min of walking time, which corresponds to the amount of walking time in patients 1 month after hospitalization for an exacerbation [7]. With a degree of certainty (statistical power) of 80 % and a risk for a type I error (α) < 5 %, 26 patients in both groups were needed. Considering a drop out rate of 20 % [34], the total sample size of the study was estimated at 62 patients with COPD.

### Study design

**Fig. 1:**
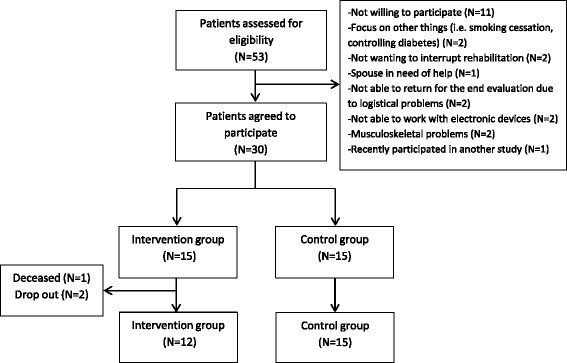
Consort diagram of the study

Fifty-three patients, hospitalized for an exacerbation in the University Hospital of Leuven, were informed about the study and eventually 30 patients were willing to participate. Patients were recruited from April 2013 to April 2014. Eligible patients were randomized (randomization rate of 1:1) into usual care or were provided with a pedometer to provide real-time feedback on physical activity and personal, telephone based PA counseling during 1 month. The intervention started from the moment of discharge. After 1 month, all patients were offered the opportunity to be enrolled in pulmonary rehabilitation. A consort diagram is provided in Fig. 1.

### Intervention

#### Physical activity counseling and real-time feedback

##### Physical activity measurement and real-time feedback

The Fitbit Ultra® (Fitbit Inc, San Francisco, California), a pedometer, was used to provide real-time feedback based on step counts. Based on a pilot study, we decided to clip the Fitbit Ultra® on the right sock in these slowly walking patients to pick up a maximal amount of steps. Because the Fitbit Ultra® is not validated in COPD, patients wore the latter device simultaneously with the Dynaport MoveMonitor (McRoberts BV, The Hague, the Netherlands), a valid activity monitor in COPD [36] at 3 time points during the study (during hospital stay, 2 weeks after discharge and at the end of the study).

##### Physical activity counseling

Telephone calls, with a frequency of 3 times a week, were used as a means to motivate and stimulate patients in the intervention group to increase their PA level during 1 month (11 ± 1 calls/patient on average). The timing of the telephone calls was determined in agreement with the patients. During these telephone contacts, step counts of the previous days were discussed with an experienced physiotherapist as well as barriers and opportunities for PA. At the end of the telephone call, a new goal was agreed for the following days. The goals were set individually, with the aim of improving the level of PA as much as possible during 1 month. Two and 4 weeks after hospital discharge, a progression report including further tips to increase PA was sent by post to the patients.

#### Usual care

Patients in the control group did not participate in any kind of rehabilitation, were not contacted nor received motivational messages. They were provided with advice about increasing PA during the hospital stay from a physiotherapist. Two weeks after hospital discharge and at the end of the study, patients were asked to wear the Dynaport MoveMonitor during 7 consecutive days.

**Fig. 2:**
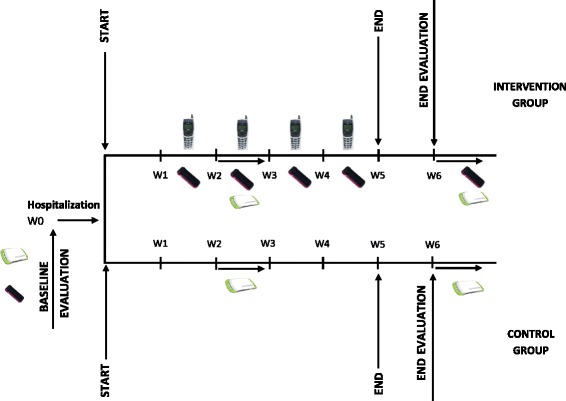
Study protocol

A more detailed description of the study protocol is depicted in Fig. 2.

### Measurements

Physical activity was the primary outcome of the study and was measured during hospital stay, 2 weeks after discharge and at the end of the study. Measurements of lung function, peripheral muscle strength, functional exercise capacity, symptoms of dyspnea and COPD-related health status were secondary outcomes of the study and were performed during hospital stay (the day before discharge) and 1 month later, at the end of the study.

#### Physical activity

The measurements were performed with the Dynaport MoveMonitor (McRoberts BV, The Hague, the Netherlands). This device was recently validated in patients with COPD [36]. The Dynaport MoveMonitor is a small (64x62x13mm) and lightweight device (68 g, including batteries). Analysis of raw data allows for classification of intensity, duration and frequency of movement. Different postures and walking are identified and energy expenditure is estimated. The Dynaport MoveMonitor is inserted in an elastic belt and positioned on the lower back at the height of the second lumbar vertebra, nearby the body’s center of mass, according to the instructions of the manufacturer. Data on walking time (PAwalk), daily amount of steps (PAsteps) and movement intensity during walking (PAint) were used for the analyses.

#### Lung function

All patients performed post-bronchodilator spirometry according to European Respiratory Society and American Thoracic Society standards [37]. The results were referred to the predicted values reported by Quanjer et al. [38].

#### Peripheral muscle strength

Isometric quadriceps strength (QF) was measured using a dynamometer (Biodex system 4 pro; Enraf Nonius; Delft, The Netherlands). Peak extension torque was evaluated at 60° of knee flexion. After a practice trial, tests were performed at least 3 times and the best result was used for further analyses [18]. Reference values for the quadriceps strength were developed in our laboratory [39].

#### Functional exercise capacity

Functional exercise performance was measured by a six minutes walking distance test (6MWD) in a 50-m corridor. The patients were instructed to walk the largest distance as possible during 6 min. Encouragements were standardized and oxygen saturation and heart rate were measured continuously. Patients included in the study were all familiar with the 6MWD. For this reason and for not burdening the patients too much, only 1 test was executed. Normal values were described by Troosters et al. [40].

#### Questionnaires

##### Modified medical research council dyspnea scale (mMRC)

The mMRC dyspnea scale is a questionnaire that consists of five statements about perceived breathlessness. Those who grade themselves with a statement with a higher score, experience more breathlessness during daily activities [41].

##### COPD assessment test (CAT)

The CAT consists of eight items, each formatted as a semantic six-point differential scale. The total score is calculated as the sum of the responses. The higher the CAT score, the lower the overall COPD-related health status. This questionnaire is valid and reliable to be used in patients with COPD [42].

### Statistical analyses

We included all evaluable patients in the statistical analyses, without excluding patients that were not compliant. Patients without follow-up data were excluded.

To check for normality, a Shapiro-Wilk test was applied. Data were expressed as mean ± SD in case of normal distribution. If the data were not normally distributed, median [IQR] were used. To compare continuous data between the two study groups an unpaired t-test was applied. The comparison of proportions between groups was performed using a Chi-Square test. Within group changes during 1 month were assessed by means of a paired t-test, using delta scores. Physical activity was measured at 3 time points. To analyze these data, a mixed model repeated measures ANOVA (proc mixed in SAS 9.3) was applied and data were corrected for important baseline differences.

## Results

### Baseline characteristics

Table 1 provides an overview of the baseline characteristics of the study. The 2 study groups were matched in terms of demographic characteristics. Functional exercise capacity was low in both the control group and the intervention group, but was significantly lower in the intervention group compared to the control group. PAsteps, PAwalk and PAint indicated an extreme physical inactivity at hospital discharge, but were not significantly different between groups.

**Table 1:** Baseline characteristics

	Control group (*N* = 15)	Intervention group (*N* = 15)	*P*-value
Demographic characteristics			
Age (years)	68 ± 6	66 ± 7	0.34
BMI (kg/m^2^)	29 ± 5	25 ± 9	0.20
Gender (male (N (%))	9 (60)	8 (53)	0.71
Pulmonary function			
FEV_1_ (% predicted)	48 ± 18	38 ± 17	0.11
Tiffeneau Index (%)	47 ± 13	41 ± 14	0.28
Peripheral muscle strength			
QF (Nm)	112 ± 28	84 ± 42	0.05
QF (% predicted)	85 ± 43	71 ± 38	0.38
Functional exercise capacity			
6MWD (meter)	317 ± 95	235 ± 134	0.07
6MWD (% predicted)	53 ± 16	36 ± 18	0.01
Stops during 6MWD (amount)	0.66 ± 0.62	1.23 ± 0.92	0.15
Duration of stops (s)	38 ± 48	81 ± 61	0.08
Physical activity			
PAsteps (amount/day)	1557 ± 1319	1644 ± 2751	0.93
PAwalk (minutes/day)	20 ± 17	22 ± 35	0.90
PAint (m/s^2^)	1.34 ± 0.50	1.46 ± 0.25	0.50
Questionnaires			
mMRC (points)	2 [2–3]	3 [2–3]	0.39
CAT (points)	19 [15–22]	25 [13–28]	0.42

*BMI* body mass index, *FEV*_*1*_ forced expiratory volume in 1 s, *QF* quadriceps strength, *6MWD* six minutes walking distance, *PAsteps* daily amount of steps, *PAwalk* daily walking time, *PAint* movement Intensity during walking, *mMRC* modified medical research council dyspnea scale, *CAT* COPD assessment test. Data are expressed as mean ± SD, median [IQR] or as N (%). *p* < 0.05

### Drop out and follow-up completers

From the intervention group, 2 patients dropped out from the study after signing the informed consent. One patient was not motivated anymore to take part, while the other patient reported transport difficulties. One patient from the intervention group deceased during the study period. The final analyses were performed including 15 patients in the control group and 12 patients in the intervention group. From the 27 patients that completed the study, a complete dataset was available.

### Change in physical activity during 1 month

**Fig. 3:**
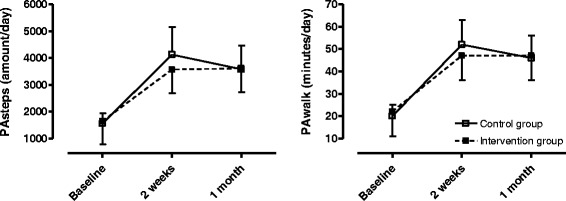
Changes in physical activity during 1 month in the 2 study groups, measured by the Dynaport MoveMonitor

**Fig. 4:**
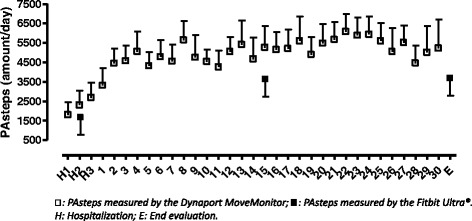
Day-by-day pattern in PAsteps measured by the Fitbit Ultra® during the intervention period

Figure 3 and Table 2 show the change in physical activity during 1 month. PAsteps and PAwalk significantly increased over time in each group, with no differences observed between groups (PAsteps: *p* = 0.96; PAwalk: *p* = 0.98). The day-by-day pattern of PAsteps measured by the Fibit Ultra during the intervention period (mean for the whole group) is shown in Fig. 4.

**Table 2:** Change in physical activity during 1 month measured by the Dynaport MoveMonitor

	Control group (*N* = 15)	Intervention group (*N* = 12)
ΔPAsteps (amount/day)		
Time effect (*p* = 0.0005)	1013 ± 1275	984 ± 1208
ΔPAwalk (minutes/day)		
Time effect (*p* = 0.0002)	13 ± 14	13 ± 16
ΔPAint (m/s^2^/day)		
Time effect (*p* = 0.07)	0.08 ± 0.06	0.06 ± 0.05

*PAsteps* daily amount of steps, *PAwalk* daily walking time, *PAint* movement Intensity during walking, Data are expressed as mean ± SD *p* < 0.05

### Change in clinical parameters during 1 month

Functional exercise capacity significantly recovered within each group 1 month after hospital discharge (Δ6MWD: control group: 64 ± 59 m (*p* = 0.02) vs intervention group: 67 ± 84 m (*p* = 0.02)). Physical activity counseling did not result in better improvements in functional exercise capacity. Muscle strength did not significantly change in each group during 1 month and was not influenced by the type of intervention. The decrease in CAT (points) was statistically and clinically [43] significant within the 2 study groups (ΔCAT: control group: −5 [−7 to 1] points (*p* = 0.02) vs intervention group: −3 [−10 to 1] points (*p* = 0.03)), with no between group effect (Table 3).

**Table 3:** Change in clinical parameters during 1 month

	Control group (*N* = 15)	Intervention group (*N* = 12)	*P*-value
Muscle strength			
ΔQF (Nm)	0.4 ± 20	5.3 ± 12	0.48
Functional exercise capacity			
Δ6MWD (meter)	64 ± 59	67 ± 84	0.93
ΔStops during 6MWD (amount)	0.08 ± 1.16	0.18 ± 1.08	0.84
ΔDuration of stops (s)	−14 ± 52	−18 ± 57	0.86
Questionnaires			
ΔmMRC (points)	0 [−1 to 0]	0 [−1 to 0]	0.93
ΔCAT (points)	−5 [−7 to 1]	−3 [−10 to 1]	0.78

*QF* quadriceps strength, *6MWD* six minutes walking distance, *mMRC* modified medical research council dyspnea scale, *CAT* COPD assessment test. Data are expressed as mean ± SD or as median [IQR]. *p* < 0.05

### Hospital readmission, medication intake during 1 month and enrollment in pulmonary rehabilitation afterwards

There was no significant difference in the amount of patients restarting oral corticosteroids during the study between the control group and the intervention group (6 (40 %) vs 5 (42 %) (*p* = 0.93)). Six patients (40 %) in the control group and 4 patients (33 %) in the intervention group were readmitted to the hospital for an acute COPD exacerbation (*p* = 0.72) within the 1 month study period. Enrollment in pulmonary rehabilitation was limited, as only 1 patient (7 %) from the control group and 3 patients (25 %) from the intervention group participated (*p* = 0.29).

## Discussion and future plans

To our knowledge, this is the first study investigating an alternative for conventional pulmonary rehabilitation after an exacerbation to increase physical activity. We investigated the use of telephone based physical activity counseling and providing patients with a pedometer and an agreed physical activity goal immediately after a hospitalization for an exacerbation. Our study showed a spontaneous but limited recovery in PA and clinical outcomes. Contrary to our expectations, counseling and real-time feedback did not result in better improvements compared to the group receiving usual care. Recovery in functional exercise capacity and COPD-related health status were similar in both groups. The baseline physical activity levels of the patients included in our study were in line with the study of Pitta et al. [7] and confirm the severe and long lasting physical inactivity during hospital admission and thereafter. In addition, the data of the pedometer in the treatment group suggest that recovery is gradual in the first 4 days but seems to level off. In contrary to our expectations, relative values of peripheral muscle strength at baseline were higher than expected in both study groups and might be explained by the presence of cachexia (BMI < 20 kg/m^2^) [44] in respectively 2 (13 %) and 3 (25 %) patients in the control and intervention group.

PA counseling and real-time feedback have been applied more successfully in stable patients with COPD [33, 34], in other chronic disease conditions [11, 30, 31] and in healthy subjects [29]. Motivational factors, physical as well as mental or social barriers to engage in PA and hospital readmission are reasons that might explain why we were unable to show that PA counseling was effective as a treatment modality in these patients. In a recent study of Greening et al. [26], a first attempt was made to investigate an alternative intervention to conventional pulmonary rehabilitation to improve function and PA during or immediately after an exacerbation. Early rehabilitation, consisting of exercise and resistance training, neuromuscular electrical stimulation and self-management was started within 48 h after the exacerbation and evolved in an unsupervised home-based program after hospital discharge. The results of this study were comparable with our study and showed a recovery in function and PA. Early rehabilitation and unsupervised home-based training did not result in a better recovery compared to usual care. The study of Greening et al. [26] therefore confirms that home based interventions to improve function or PA during or immediately after hospital admission for an exacerbation might lack efficacy. Individualized pulmonary rehabilitation after an exacerbation may be a better approach in patients after an exacerbation. We speculate that the current intervention could be an alternative for patients that explicitly express a willingness to increase PA, but cannot access a pulmonary rehabilitation program. Motivation to alter PA was not an inclusion criterion and unfortunately, motivational aspects were not registered in the current study. One of the limitations of our study was that we did not take into account “motivation to alter PA” as an inclusion criterion and that motivational aspects were not registered. Further, we did not use a more interactive platform, as was applied by Moy et al. [34]. The majority of our patients (70 %), however, did not have internet access and although this might rapidly change in the future, this seems currently not to be an option. We did not have a rewarding system for people that effectively increased PA in order to provide extrinsic motivation and we did not combine the intervention with home visits to overcome practical barriers for physical inactivity and to explore concrete solutions with the patient. The last limitation of our study was that we did not spread the intervention over a longer time window to allow a slower continued recovery. The fact that patients seemed to stagnate in their PA levels, however, suggests that not much further improvements should be expected by simply prolonging the present intervention. In order to improve the effectiveness of future studies, a rewarding system, home visits and the investigation of motivational aspects should be implemented.

Although the low sample size of the present study limits the generalizability of the data, we concluded that PA counseling immediately after an exacerbation is time consuming and not effective to enhance PA or clinical outcomes. Individualized pulmonary rehabilitation in the period after an exacerbation of COPD might be a better approach.

### Future plans

By executing this study, we experienced that including patients after an exacerbation of COPD is very difficult. After 1 year, only 30 patients agreed to participate in the study. Intermediate analyses did not reveal better improvements in physical activity in the group that received maximal counseling and real-time feedback compared to usual care. Based on these conclusions and the fact that the study was very time consuming for the researcher, we decided not to continue with the study.

## Conclusion

We concluded that physical activity levels, functional exercise capacity and COPD-related health status recover 1 month after an exacerbation, but that recovery is limited. Real-time feedback and physical activity counseling is timing consuming and did not result in better improvements in comparison to usual care.

## Abbreviations

6MWD: 6 min walking distance test
CAT: COPD assessment test
COPD: Chronic Obstructive Pulmonary Disease
mMRC: modified medical research council dyspnea scale
PA: physical activity
PAint: movement intensity during walking
PAsteps: daily amount of steps
PAwalk: daily walking time
QF: quadriceps strength

## References

[1] Decramer M, Janssens W, Miravitlles M (2012). Chronic obstructive pulmonary disease. Lancet.

[2] Rabe KF, Hurd S, Anzueto A, Barnes PJ, Buist SA, Calverley P (2007). Global strategy for the diagnosis, management, and prevention of chronic obstructive pulmonary disease: GOLD executive summary. Am J Respir Crit Care Med.

[3] Roca M, Verduri A, Corbetta L, Clini E, Fabbri LM, Beghe B (2013). Mechanisms of acute exacerbation of respiratory symptoms in chronic obstructive pulmonary disease. Eur J Clin Invest.

[4] Rodriguez-Roisin R (2006). COPD exacerbations.5: management. Thorax.

[5] Wedzicha JA, Seemungal TA (2007). COPD exacerbations: defining their cause and prevention. Lancet.

[6] Burtin C, Decramer M, Gosselink R, Janssens W, Troosters T (2011). Rehabilitation and acute exacerbations. Eur Respir J.

[7] Pitta F, Troosters T, Probst VS, Spruit MA, Decramer M, Gosselink R (2006). Physical activity and hospitalization for exacerbation of COPD. Chest.

[8] Thorpe O, Kumar S, Johnston K (2014). Barriers to and enablers of physical activity in patients with COPD following a hospital admission: a qualitative study. Int J Chron Obstruct Pulmon Dis.

[9] Garcia-Aymerich J, Lange P, Benet M, Schnohr P, Anto JM (2006). Regular physical activity reduces hospital admission and mortality in chronic obstructive pulmonary disease: a population based cohort study. Thorax.

[10] Waschki B, Kirsten A, Holz O, Muller KC, Meyer T, Watz H (2011). Physical activity is the strongest predictor of all-cause mortality in patients with COPD: a prospective cohort study. Chest.

[11] Vaes AW, Cheung A, Atakhorrami M, Groenen MT, Amft O, Franssen FM (2013). Effect of ‘activity monitor-based’ counseling on physical activity and health-related outcomes in patients with chronic diseases: a systematic review and meta-analysis. Ann Med.

[12] Watz H, Pitta F, Rochester CL, Garcia-Aymerich J, ZuWallack R, Troosters T (2014). An official European Respiratory Society statement on physical activity in COPD. Eur Respir J.

[13] Coronado M, Janssens JP, de Muralt B, Terrier P, Schutz Y, Fitting JW (2003). Walking activity measured by accelerometry during respiratory rehabilitation. J Cardiopulm Rehabil.

[14] Dallas MI, McCusker C, Haggerty MC, Rochester CL, ZuWallack R (2009). Using pedometers to monitor walking activity in outcome assessment for pulmonary rehabilitation. Chron Respir Dis.

[15] Egan C, Deering BM, Blake C, Fullen BM, McCormack NM, Spruit MA (2012). Short term and long term effects of pulmonary rehabilitation on physical activity in COPD. Respir Med.

[16] Mador MJ, Patel AN, Nadler J (2011). Effects of pulmonary rehabilitation on activity levels in patients with chronic obstructive pulmonary disease. J Cardiopulm Rehabil Prev.

[17] Mercken EM, Hageman GJ, Schols AM, Akkermans MA, Bast A, Wouters EF (2005). Rehabilitation decreases exercise-induced oxidative stress in chronic obstructive pulmonary disease. Am J Respir Crit Care Med.

[18] Pitta F, Troosters T, Probst VS, Langer D, Decramer M, Gosselink R (2008). Are patients with COPD more active after pulmonary rehabilitation?. Chest.

[19] Sewell L, Singh SJ, Williams JE, Collier R, Morgan MD (2005). Can individualized rehabilitation improve functional independence in elderly patients with COPD?. Rev Port Pneumol.

[20] Steele BG, Belza B, Hunziker J, Holt L, Legro M, Coppersmith J (2003). Monitoring daily activity during pulmonary rehabilitation using a triaxial accelerometer. J Cardiopulm Rehabil.

[21] Steele BG, Belza B, Cain KC, Coppersmith J, Lakshminarayan S, Howard J (2008). A randomized clinical trial of an activity and exercise adherence intervention in chronic pulmonary disease. Arch Phys Med Rehabil.

[22] Walker PP, Burnett A, Flavahan PW, Calverley PM (2008). Lower limb activity and its determinants in COPD. Thorax.

[23] Jones SE, Green SA, Clark AL, Dickson MJ, Nolan AM, Moloney C (2014). Pulmonary rehabilitation following hospitalisation for acute exacerbation of COPD: referrals, uptake and adherence. Thorax.

[24] Arnold E, Bruton A, Ellis-Hill C (2006). Adherence to pulmonary rehabilitation: a qualitative study. Respir Med.

[25] Garrod R, Marshall J, Barley E, Jones PW (2006). Predictors of success and failure in pulmonary rehabilitation. Eur Respir J.

[26] Greening NJ, Williams JE, Hussain SF, Harvey-Dunstan TC, Bankart MJ, Chaplin EJ (2014). An early rehabilitation intervention to enhance recovery during hospital admission for an exacerbation of chronic respiratory disease: randomised controlled trial. BMJ.

[27] Taylor R, Dawson S, Roberts N, Sridhar M, Partridge MR (2007). Why do patients decline to take part in a research project involving pulmonary rehabilitation?. Respir Med.

[28] Breyer MK, Breyer-Kohansal R, Funk GC, Dornhofer N, Spruit MA, Wouters EF (2010). Nordic walking improves daily physical activities in COPD: a randomised controlled trial. Respir Res.

[29] Recio-Rodriguez JI, Martin-Cantera C, Gonzalez-Viejo N, Gomez-Arranz A, Arietaleanizbeascoa MS, Schmolling-Guinovart Y (2014). Effectiveness of a smartphone application for improving healthy lifestyles, a randomized clinical trial (EVIDENT II): study protocol. BMC Public Health.

[30] Butler L, Furber S, Phongsavan P, Mark A, Bauman A (2009). Effects of a pedometer-based intervention on physical activity levels after cardiac rehabilitation: a randomized controlled trial. J Cardiopulm Rehabil Prev.

[31] Guiraud T, Granger R, Gremeaux V, Bousquet M, Richard L, Soukarie L (2012). Telephone support oriented by accelerometric measurements enhances adherence to physical activity recommendations in noncompliant patients after a cardiac rehabilitation program. Arch Phys Med Rehabil.

[32] De Blok BM, de Greef MH, ten Hacken NH, Sprenger SR, Postema K, Wempe JB (2006). The effects of a lifestyle physical activity counseling program with feedback of a pedometer during pulmonary rehabilitation in patients with COPD: a pilot study. Patient Educ Couns.

[33] Hospes G, Bossenbroek L, ten Hacken NH, van Hengel P, de Greef MH (2009). Enhancement of daily physical activity increases physical fitness of outclinic COPD patients: results of an exercise counseling program. Patient Educ Couns.

[34] Moy ML, Janney AW, Nguyen HQ, Matthess KR, Cohen M, Garshick E (2010). Use of pedometer and internet-mediated walking program in patients with chronic obstructive pulmonary disease. J Rehabil Res Dev.

[35] Pitta F, Troosters T, Spruit MA, Probst VS, Decramer M, Gosselink R (2005). Characteristics of physical activities in daily life in chronic obstructive pulmonary disease. Am J Respir Crit Care Med.

[36] Van Remoortel H, Raste Y, Louvaris Z, Giavedoni S, Burtin C, Langer D (2012). Validity of six activity monitors in chronic obstructive pulmonary disease: a comparison with indirect calorimetry. PLoS One.

[37] Miller MR, Hankinson J, Brusasco V, Burgos F, Casaburi R, Coates A (2005). Standardisation of spirometry. Eur Respir J.

[38] Quanjer PH, Tammeling GJ, Cotes JE, Pedersen OF, Peslin R, Yernault JC (1993). Lung volumes and forced ventilatory flows. Report Working Party Standardization of Lung Function Tests, European Community for Steel and Coal. Official Statement of the European Respiratory Society. Eur Respir J Suppl.

[39] Decramer M, Lacquet LM, Fagard R, Rogiers P (1994). Corticosteroids contribute to muscle weakness in chronic airflow obstruction. Am J Respir Crit Care Med.

[40] Troosters T, Gosselink R, Decramer M (1999). Six minute walking distance in healthy elderly subjects. Eur Respir J.

[41] Bestall JC, Paul EA, Garrod R, Garnham R, Jones PW, Wedzicha JA (1999). Usefulness of the Medical Research Council (MRC) dyspnoea scale as a measure of disability in patients with chronic obstructive pulmonary disease. Thorax.

[42] Jones PW, Harding G, Berry P, Wiklund I, Chen WH, Kline LN (2009). Development and first validation of the COPD assessment test. Eur Respir J.

[43] Kon SS, Canavan JL, Jones SE, Nolan CM, Clark AL, Dickson MJ (2014). Minimum clinically important difference for the COPD assessment test: a prospective analysis. Lancet Respir Med.

[44] Evans WJ, Morley JE, Argiles J, Bales C, Baracos V, Guttridge D (2008). Cachexia: a new definition. Clin Nutr.

